# Peri-Operative Changes of Inflammatory Markers and Their Implications in Pulmonary Endarterectomy

**DOI:** 10.31083/j.rcm2311357

**Published:** 2022-10-25

**Authors:** Qianqian Liu, Ziru Zhao, Jing Yang, Yunshan Cao, Min Zhang

**Affiliations:** ^1^School of Basic Medicine, Gansu University of Chinese Medicine, 730000 Lanzhou, Gansu, China; ^2^Department of Scientific Research Office, Gansu Provincial Hospital, 730000 Lanzhou, Gansu, China; ^3^Department of Pathology, The 940th Hospital of Joint Logistics Support Force of Chinese People's Liberation Army, 730050 Lanzhou, Gansu, China; ^4^Department of Cardiology, Gansu Provincial Hospital, 730000 Lanzhou, Gansu, China; ^5^Department of Scientific Research Office, Gansu Provincial Hospital, 730000 Lanzhou, Gansu, China

**Keywords:** inflammation, pulmonary endarterectomy, perioperative period

## Abstract

Pulmonary endarterectomy (PEA) is used to treat chronic thromboembolic pulmonary hypertension (CTEPH) patients, and it can effectively remove organized thrombotic materials and proliferative intima as well as improve hemodynamics. It has been reported that the levels of several inflammatory factors were altered in the peri-operative period of PEA. Even though their specific role remains unknown, this could have some relevance. In this study, we reviewed the recently published data addressing these factors in PEA, attempting to understand their potential implications.

## 1. Introduction 

Pulmonary endarterectomy (PEA) surgery has been the primary treatment option for patients suffering from chronic thromboembolic pulmonary hypertension (CTEPH) [1]. PEA was first proposed and popularized by a team from the University of California in San Diego, USA [2]. PEA surgery can clear organized thromboembolic materials and enhance the hemodynamic index of pulmonary circulation and prognosis among CTEPH patients [3, 4]. However, some potential complications are causing early postoperative mortality rates of 5%–23% [5]. It has been reported that PEA-associated hemodynamic instability during the perioperative period was usually correlated with cytokines overstimulation [6]. Additionally, several studies have revealed that the blood samples of CTEPH patients after PEA surgery contained a lot of inflammatory factors and cytokines [5, 7, 8]. With an increasing number of reports regarding PEA inflammation, it is essential to review recent data for further understanding the implications of PEA that are beneficial for patients.

## 2. CTEPH-Related 

Inflammation involves the pathogenesis of CTEPH and has a critical role in the process of right heart failure. Many studies identified high levels of inflammatory cytokines in the blood samples of CTEPH patients, such as interleukin (IL)-6, tumor necrosis factor (TNF)-α, c-reaction protein (CRP), etc. [9]. A previous study reported that the levels of IL-1β, IL-6, IL-8, and TNF-α levels were higher in CTEPH endothelial cells than in the control groups [10]. These changes in cytokines could indicate the relevance of the prognosis of CTEPH patients during the perioperative stage of PEA surgery. The levels of inflammatory mediators varied at different time points after PEA surgery (Table 1A,1B,1C, Ref. [5, 6, 7, 11, 12, 13, 14]). Thus, well-studied cytokines were introduced as follows.

**Table 1A.** **The level of inflammation cytokines after PEA surgery**.

Author	Samples	TNF-α		IL-6		IL-8	IL-10	
		Preoperative	Postoperative	Preoperative	Postoperative		Preoperative	Postoperative
Langer F *et al*. (2004) [5]	14	Elevated in 8 patients	They decreased significantly within 24 h after PEA surgery in 12 patients	Elevated in 5 patients	A sharp peak immediately after surgery, decreased during the postoperative period but was higher than the baseline	-	Elevated in 4 patients	During surgery, the level increased significantly, followed by a drastic decrease. After 8 h of surgery, it reached its baseline levels
Maruna P *et al*. (2008) [11]	32	Reached its peak after 24 h sternotomy, returned to preoperative level after 48 h sternotomy		Elevated in 6 of 32 patients 24 h before surgery.	The peak level of IL-6 was 6 h after sternotomy. The level was higher than preoperative after 48 h sternotomy	Reached its peak 12 h after sternotomy. The level was higher than preoperative after 48 h sternotomy	-	-
Lindner J *et al*. (2009) [6]	36	Elevated at the time of separation from CPB, returned to preoperative level after 48 h CPB		Elevated at the time of separation from CPB, the level was also higher than the preoperative level after 48 h CPB		Reached its peak 12 h after CPB, which was also higher than the preoperative level after 48 h CPB	-	-
Maruna P *et al*. (2009) [14]	32	Reached its peak 6 h after CPB		Reached its peak 6 h after CPB		Reached its peak 12 h after CPB	-	-
Maruna P *et al*. (2009) [12]	22	Reached its peak 12 h after CPB		Reached its peak 12 h after CPB, the level was higher than preoperative after 36 h CPB		Reached its peak 18 h after CPB	-	-
Maruna P *et al*. (2011) [7]	82	The level elevated after surgery		Reached its peak 12 h after CPB, then fall		-	-	-
Maruna P *et al*. (2011) [13]	24	Reached its peak 12 h after CPB		Reached its peak 12 h after CPB, then fall		Reached its peak 18 h after CPB	-	-

**Table 1B.** **The level of inflammation cytokines after PEA surgery**.

Author	Samples	IL-1β	C-reactive protein	Procalcitonin		Leptin	Soluble leptin receptor
				Preoperative	Postoperative		
Maruna P *et al*. (2008) [11]	32	Elevated 6 h after surgery, not have statistically significant	Elevated 12 h after sternotomy, reached a peak level 48 h after sternotomy	Normal	Transient initial decline 3 h after sternotomy, reached a peak level 24 h after sternotomy. The level was higher than preoperative after 48 h sternotomy	-	-
Lindner J *et al*. (2009) [6]	36	Elevated at the time of separation from CPB, returned to preoperative level after 48 h CPB	-	-	-	-	-
Maruna P *et al*. (2009) [14]	32	Elevated 6 h after surgery, not significantly different from the initial levels	-	-	-	Transient initial decline 3 h after sternotomy, reached a peak level 24 h after sternotomy. The level was higher than preoperative after 48 h after sternotomy	Transient initial decline 3 h after sternotomy, returned to initial level 24 h after surgery
Maruna P *et al*. (2009) [12]	22	Elevated 6 h after surgery, not statistically significant	Elevated with a peak level at 48 h after CPB. The level was higher than preoperative after CPB 72 h	-	-	-	-
Maruna P *et al*. (2011) [7]	82	-	Elevated with a peak level at 48 h after CPB. The level was higher than preoperative after CPB 72 h	Minimal PCT concentrations were found after the last DHCA, reaching a peak level 24 h after the end of surgery. The level was higher than preoperative after CPB 72 h		-	-
Maruna P *et al*. (2011) [13]	24	-	Elevated with a peak level at 48 h after CPB	-	-	-	-

**Table 1C.** **The level of inflammation cytokines after PEA surgery**.

Author	Samples	Cortisol	Hepcidin	Pro-hepcidin
Maruna P *et al*. (2009) [14]	32	Reached its peak 6 h after sternotomy, remained elevated 48 h after the start of surgery	-	-
Maruna P *et al*. (2009) [12]	22	-	-	The initial decline after DHCA reached its minimal after CPB, returned to initial levels within 24–48 h after the separation from CPB
Maruna P *et al*. (2011) [7]	82	-	-	-
Maruna P *et al*. (2011) [13]	24	-	Elevated from the start of surgery to 72 h after surgery, but the level was higher than preoperative 120 h after surgery	-

Tumor Necrosis Factor-alpha (TNF-α) is an inflammatory cytokine that was upregulated in the blood of CTEPH patients as compared to the control groups. It has been reported that increasing levels of TNF-α could indicate the extent of right heart failure associated with CTEPH [5]. Though the preoperative TNF-α levels were high, there was no relation to the preoperative pulmonary hemodynamic status. After PEA surgery, the expression of TNF-α was significantly downregulated [5]. In their study, two patients died when the TNF-α levels peaked after PEA surgery and had persistent postoperative pulmonary hypertension during the postoperative period [5]. One study indicated that CTEPH patients had more severe nocturnal hypoxia than idiopathic pulmonary hypertension patients, and the nocturnal mean ${\mathrm{SpO}}_{2}$ was an independent risk factor for high TNF-α levels. From a long perspective, high levels of TNF-α induced by nocturnal hypoxia could exacerbate the degrees of CTEPH [15]. TNF-α could improve pulmonary vascular reactivity and promote pulmonary vasoconstriction induced by platelet-activating factors in animal models [16]. Furthermore, TNF-α can promote pulmonary vascular smooth cell proliferation through bone morphogenetic protein type-II receptor signaling [17].

Upregulated IL-6 could not correlate with the hemodynamic status pre-operation [11]. Since PEA surgery could inflict significant damage to the pulmonary vascular endothelium, the release of IL-6 originates from the pulmonary vascular endothelium [18]. Therefore, IL-6 expression could be caused by systemic vasoplegia in CTEPH patients after PEA [5]. It has also been described that postoperative IL-6 significantly correlated with preoperative mean pulmonary artery pressure and pulmonary vascular resistance levels of CTEPH [5]. Although the IL-6 levels increased and peaked at 6–12 h after the operation and then decreased, they were still higher than before the procedure [5, 6, 7, 11, 12, 13]. Moreover, several studies revealed that IL-6 significantly correlated with the vasopressor (norepinephrine) [6, 13]. This may indicate that cytokine activation may be neurohumoral, which is responsible for the hemodynamic changes in CTEPH patients after PEA [6]. IL-6 also correlates with the proliferation of pulmonary vascular smooth cells [19] and is essential in pulmonary vascular remodeling, which induces pro-inflammatory and pro-angiogenic transcriptional programs by activating the janus kinase (JAK) pathways and signal transducer and activator of transcription (STAT) signaling [20]. Additionally, IL-6 also correlates with right ventricular function [21]. The IL-6 knockout model depicted decreased cardiac hypertrophy, fibrosis, and inflammation after angiotensin II stimulation [22]. Another study pointed out that an increased level of IL-6 can predict residual pulmonary hypertension after PEA surgery [23]. Numerous members of the IL-6 family and their levels also change during the perioperative period.

C-reactive protein (CRP), an acute-phase hepatic protein, immediately responds to inflammatory reactions within the disease organs [24]. Furthermore, high levels of CRP negatively correlated with the 6-minute walk test distance and right ventricular (RV) function in a sizeable CTEPH cohort [25]. In addition, CRP levels ≥10 mg/L were associated with death or the requirement for lung transplantation during the 57 months’ follow-up period. Suppose preoperative CRP concentration was higher than 10 mg/L in CTEPH patients, it may be associated with poor early outcomes after PEA surgery. Thus, elevated plasma levels of CRP were associated with severe postoperative hemodynamics in CTEPH patients after PEA surgery [26]. The plasma levels of CRP are increasing in response to inflammation, and their expression is mainly correlated with IL-6, and to a lesser extent interleukin-1, TNF-α, macrophages, and T-cells [27]. CRP promotes vascular remodeling and pulmonary endothelial dysfunction by enhancing the pulmonary arterial smooth vascular cell mitogenic activity and monocyte adhesion to pulmonary arterial endothelial cells, endothelin (ET)-1, and von Willebrand factor (vWF) secretion among CTEPH patients [28].

IL-8 is an effective pro-inflammatory cytokine that is released by endothelial cells, monocytes, T cells, etc [29]. It has been reported that high baseline levels of IL-8 are negatively associated with the survival of CTEPH patients (but not all CTEPH patients had high levels of IL-8 at baseline) [30]. The changes in IL-8 occurred slightly later than in IL-6 (Table 1A,1B,1C). IL-8 levels significantly correlated with the levels of cardiac Troponin I. It indicated that IL-8 plays a role in cardiac injury after cardiac surgery [31]. IL-8 can elevate the adhesion between cells and increase the infiltration of neutrophils [32]. It has also been described that IL-8 is associated with the hemodynamic status after PEA surgery, whose concentration reaches its peak 12 h after separation from the cardiopulmonary bypass (CPB) [6].

IL-10 as an anti-inflammatory cytokine can prevent inflammatory cell infiltration and smooth muscle cell proliferation in patients with pulmonary hypertension [33]. It has been revealed that IL-10 reduces cardiac hypertrophy and fibrosis and protects cardiac function and vasculature [22]. IL-10 expression also elevated reactively preoperative along with the elevated levels of TNF-α, and the IL-10 levels peaked immediately after surgery. This may be a concomitant pro-inflammatory and anti-inflammatory cytokine response [5]. The release of IL-10 could indirectly prevent the ongoing production of pro-inflammatory cytokines [14].

## 3. Procedure-Related 

**Fig. 1.**
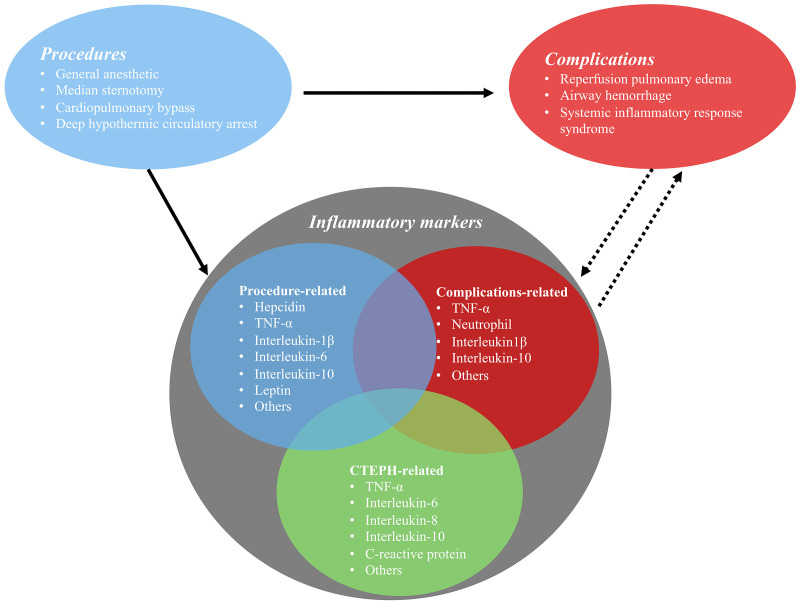
**The relationship between pulmonary endarterectomy and perioperative inflammatory markers**.

PEA surgery cures CTEPH patients, and the survival rates reach 90% after five years of surgery [34]. PEA surgery includes a general anesthetic, median sternotomy, CPB, and deep hypothermic circulatory arrest (DHCA) [35, 36]. These procedures had been to be reported to affect the immune system [6, 37] and contributed to the increasing levels of inflammatory cytokines (Fig. 1).

CPB results in a hemodynamic state of loss of pulsatile flow and micro-embolism [38]. Blood exposure to CPB circuits leads to the systemic inflammatory response, leukocyte activation, and the release of pro-inflammatory and anti-inflammatory cytokines [39], ultimately causing multi-organ function failure. The CPB of PEA surgery was performed with a non-pulsatile flow [40]. Studies have shown that non-pulsatile perfusion causes a decrease in hemodynamic energy, resulting in capillary collapse, microvascular shunting, and the activation of inflammatory mediators [40, 41]. Another study compared the centrifugal pump and roller pump induced inflammatory cytokines in CPB after PEA surgery. It was concluded that the non-occlusive centrifugal pump was associated with reduced inflammation [42]. These findings warrant further studies exploring the changes in inflammatory cytokines after CPB during PEA surgery. Elevated levels of IL-10 were associated with pre-operative steroid injections in CPB surgery, which reduced myocardial injury [43], attenuated the inflammatory response to cardiac surgery and CPB, and enhanced hemodynamic changes [44]. IL-1β is also a pro-inflammatory cytokine, and its levels were upregulated at the time of separation from CPB and returned to the preoperative level after 48 h of CPB [6]. In other studies, the levels of IL-1β elevated at 6h after surgery but were not significantly different from the preoperative condition [11, 12, 14]. The role of this cytokine in postoperative PEA surgery requires further investigation. IL-1β and TNF-α contribute to the proliferation of pulmonary vascular smooth cells and thrombosis [45]. In addition, IL-1β promoted inflammatory infiltrates in pulmonary arteries [45]. Hepcidin, a type II acute-phase protein, can predict the risk of acute kidney injury after CPB [46], leading to increased mortality and hospitalization [47]. The changes in hepcidin could control the availability of iron microorganisms during the infection [48]. Elevated concentration of postoperative hepcidin correlated with the increasing levels of IL-6 post-surgery and reached its peak 72 h after the separation from CPB [13].

DHCA provides surgeons with a relatively clear view to allow for precise dissection and removal of obstructive material during PEA surgery [49]. Neurological injury is the most common complication caused by DHCA [50]. It has been reported that the incidence of perioperative neurological injury of PEA was within the range of 3%–12% [50, 51, 52]. The longer time of DHCA may inflict neurological injury [53] and acute kidney injury [54]. Surgeons should remove the thrombosis materials as soon as possible to minimize the times and duration of the circulatory arrest. In the PEACOG (PEA and COGnition) trial, the affection of PEA to cognitive function was investigated, and data revealed that PEA with DHCA at 20 °C provided excellent lung and brain results using other standard procedures [51]. DHCA also contributed to the release of inflammatory cytokines such as TNF-α and IL-6 [55].

Anesthetics are critical to the surgical procedure. It is a challenge for anesthesiologists to use anesthetics in CTEPH patients during PEA surgery due to the risk of right heart failure [56]. Sevoflurane is used in CTEPH (PEA) patients because of its cardio-protective effects in patients with little or no ischemic heart disease as a result [57]. It has been reported that anesthetics can regulate the immune system, including apoptosis of lymphocytes and impairment of neutrophil phagocytosis [58], which could be involved in PEA surgery.

Surgery-related trauma can specifically induce neutrophil extracellular trap formation that causes endothelial cell damage and impairment of vascular integrity, eventually resulting in postoperative multi-organ function failure [59]. High levels of IL-6 and IL-10 induced by surgical-related trauma correlated with the incidence of multi-organ function failure and mortality [60]. Furthermore, the increased levels of IL-6 during the perioperative period can impair cognitive function during the postoperative phase [61]. However, inflammatory cytokines are not only detrimental to cardiac remodeling, sometimes they can play protective roles in cardiac remodeling. For example, IL-6 plays a protective role in the heart by activating glycoprotein (GP)130 [62], thereby leading to a cellular protective response in the heart, which preserves cellular and interstitial structural integrity [63]. Alterations in the immune system induced by surgical trauma correlated with postoperative recovery [59]. Leptin is similar to other acute phase reactants and can upregulate the levels of proinflammatory cytokines such as TNF-α, and IL-6 [64]. After PEA surgery, the levels of leptin were significantly elevated at 24 h after sternotomy and decreased 48 h after surgery. The following decrease in leptin levels does not correlate with the further insult that can produce more leptin production [14]. Synergistic effects of local or systemic TNF or IL-6 combined with glucocorticoids may contribute to increased leptin expression in response to surgical stress [14]. Further studies are required about PEA surgery *per se* induced inflammatory responses.

## 4. Complications-Related 

The most common complications of PEA surgery include reperfusion pulmonary edema [37], airway hemorrhage, and systemic inflammatory response syndrome [65] (Fig. 1). Inflammation is critical in these processes. Pulmonary hemorrhage is a rare and serious complication and is associated with a mortality rate approaching 70% [66]. This can be caused by tears or disruption in the intima of the pulmonary artery (surgical struma), bleeding due to the rupture of fragile bronchopulmonary collateral, damage to the blood-airway barrier, and reperfusion pulmonary edema [67]. Other risk factors correlated with hemorrhage have not been investigated thoroughly.

Reperfusion lung injury is common among PEA patients and usually leads to postoperative morbidity and mortality [68, 69]. Reperfusion injury is a specific complication that usually appears within 48 h after surgery, which is similar to acute lung injury. The main characteristic is pulmonary hyperemia in the revascularized pulmonary areas [70]. It has previously been reported that the neutrophil could induce the reperfusion of lung injury [71]. It has been demonstrated that increased neutrophils exist in bronchial alveolar lavage of patients with lung injury compared with those without lung injury [72]. Blocking the neutrophil selectin-mediated adhesion on the day of surgery reduced the reperfusion injury incidence [72]. These findings suggested that inflammation could induce reperfusion injury. Also, pulmonary reperfusion after PEA surgery made patients more susceptible to infection [7]. Administration of cylexin to CTEPH patients undergoing PEA reduces the incidence of postoperative reperfusion lung injury. However, no significant effect was observed on the overall clinical outcome [72]. In the latest study, predictively injecting erythropoietin to patients with CPB surgery effectively reduced lung injury after CPB and reduced the pro-inflammatory factors TNF-α and IL-1β after CPB, and also promoted the release of the anti-inflammatory factor IL-10 [73]. This may give us a clue whether injecting erythropoietin in CTEPH patients after PEA surgery is possible. Further efforts and studies are required to explore the ways to reduce lung injury after PEA surgery. Extravascular lung water measurements at 24 h and 36 h after surgery can provide an accurate diagnosis of severe reperfusion injury. These data are clinically useful to clinicians to provide corresponding treatments [74].

Residual pulmonary hypertension is the most common reason for postoperative mortality and morbidity in CTEPH patients after PEA surgery [52, 75]. It results from distal chronic thromboembolic disease or small-vessel vasculopathy that is not cured by endarterectomy [76]. Within the international CTEPH registry, persistent pulmonary hypertension affected 16.7% of patients and was related to a higher early mortality rate [52]. In addition, patients can also re-present with CTEPH and pulmonary hypertension, which is caused by a further thrombotic episode after successful PEA [3, 77] and usually correlates with irregularly anticoagulation [3]. Until now, there is no clear guidance for CTEPH patients undergoing PEA surgery to detect recurrent pulmonary hypertension [77].

## 5. Others

It has been reported that preoperative parenchymal lung disease decreased the perioperative pulmonary reserve and was a predictor of perioperative mortality after PEA [78]. A small study (86 cases) revealed that the incidence of prolonged mechanical ventilation was nearly 50% after PEA surgery, which also correlated with higher rates of postoperative complications and higher hospital medical expenses [79]. Prolonged tracheal intubation is common after PEA. Thus, it is expected that ventilator-associated pneumonia would be prevalent in CTEPH patients after PEA [78]. Furthermore, a longer time of ventilatory support and a longer stay in the intensive care unit would increase the possibility of nosocomial or care-related infections, leading to death [80]. Procalcitonin (PCT) is a highly specific marker for the diagnosis of clinically relevant bacterial infections and sepsis [11], and is used as a predictive factor for postoperative complications following cardiac surgery [81, 82]. Plasma levels of PCT are elevated due to the systemic inflammatory responses [83] and act as a predictive factor in distinguishing inflammatory and noninflammatory complications. The expression of PCT reaches its peak at 24 h after the end of the surgery, along with the downregulated pro-inflammatory mediators such as IL-6 and TNF-α [7]. In non-infected patients, PCT peaked at 24 h after the end of the surgery and decreased to half its peak values on the following day [7]. The subsequent decline in PCT levels after PEA surgery correlated with the half-life of PCT (18–24 h), indicating the absence of a further insult that could induce the increasing level of PCT production [7]. Together, these results revealed that PCT and IL-6 significantly contributed to identifying an infection after PEA [7].

## 6. Conclusions

Pulmonary endarterectomy is an effective treatment for chronic thromboembolic pulmonary hypertension patients. Perioperative inflammatory cytokines are crucial to improve the prognosis of patients. Furthermore, the existence of inflammatory mediators in removed PEA materials could further suggest that inflammatory cytokines are involved in the pathogenesis of CTEPH. In a word, the exact role of involved inflammation peri-PEA remains unknown and should be explored in the future.

## Abbreviations

PEA, Pulmonary endarterectomy; CTEPH, Chronic thromboembolic pulmonary hypertension; CPB, Cardiopulmonary bypass; DHCA, Deep hypothermic circulatory arrest; TNF-α, Tumour Necrosis Factor-alpha; IL-6, Interleukin-6; IL-8, Interleukin-8; IL-10, Interleukin-10; JAK, Janus kinase; STAT, Signal transducer and activator of transcription; PCT, Procalcitonin; CRP, C-reactive protein; ET-1, Endothelin-1; vWF, Von Willebrand factor; IL-1β, Interleukin-1β.
